# Signaling Through gp130 Compromises Suppressive Function in Human FOXP3^+^ Regulatory T Cells

**DOI:** 10.3389/fimmu.2019.01532

**Published:** 2019-07-18

**Authors:** Khalid Bin Dhuban, Sabrina Bartolucci, Eva d'Hennezel, Ciriaco A. Piccirillo

**Affiliations:** ^1^Department of Microbiology and Immunology, McGill University, Montreal, QC, Canada; ^2^Program in Infectious Diseases and Immunology in Global Health, Centre for Translational Biology, Research Institute of the McGill University Health Centre, Montreal, QC, Canada; ^3^Centre of Excellence in Translational Immunology, Montreal, QC, Canada

**Keywords:** FOXP3, regulatory T cell, suppression, autoimmunity, immune regulation, gp130, cytokines

## Abstract

The CD4^+^FOXP3^+^ regulatory T cell (Treg) subset is an indispensable mediator of immune tolerance. While high and stable expression of the transcription factor FOXP3 is considered a hallmark feature of Treg cells, our previous studies have demonstrated that the human FOXP3^+^ subset is functionally heterogeneous, whereby a sizeable proportion of FOXP3^+^ cells in healthy individuals have a diminished capacity to suppress the proliferation and cytokine production of responder cells. Notably, these non-suppressive cells are indistinguishable from suppressive Treg cells using conventional markers of human Treg. Here we investigate potential factors that underlie loss of suppressive function in human Treg cells. We show that high expression of the IL-6 family cytokine receptor subunit gp130 identifies Treg cells with reduced suppressive capacity *ex vivo* and in primary FOXP3^+^ clones. We further show that two gp130-signaling cytokines, IL-6 and IL-27, impair the suppressive capacity of human Treg cells. Finally, we show that gp130 signaling reduces the expression of the transcription factor Helios, whose expression is essential for stable Treg function. These results highlight the role of gp130 in regulating human Treg function, and suggest that modulation of gp130 signaling may serve as a potential avenue for the therapeutic manipulation of human Treg function.

## Introduction

CD4^+^FOXP3^+^ regulatory T cells (Treg) play an essential role in the maintenance of tolerance to self and harmless antigens. Congenital or acquired deficiencies in Treg cells result in severe autoimmunity in several animal models as well as in humans, and adoptive transfer of Treg cells controls autoimmunity in several animal models (1, 2). Numerous studies have examined potential defects in the Treg population as underlying or contributory factors in human organ-specific autoimmunity [reviewed in (2)]. While several groups reported numerical and functional defects in the Treg compartment in a number of autoimmune diseases such as multiple sclerosis (MS), type 1 diabetes (T1D), rheumatoid arthritis (RA) and systemic lupus erythematosus (SLE), others have observed normal Treg frequency and function in these diseases [reviewed in (2)]. In addition to potential disease heterogeneity and methodological variations that may have contributed to the variable findings in these studies, lack of reliable human Treg cell markers is a significant limitation (2). While a number of markers allow for the detection of highly-enriched Treg cells in resting conditions, most of these markers are inducible on effector T cells (Teff) upon TCR-mediated activation, thus leading to the inclusion of activated Teff contaminants and increasing the functional heterogeneity of the population (2). This results in considerable phenotypic and functional variability in the global Treg cell pool examined from healthy individuals and autoimmune patients, thus confounding the interpretation of results. Furthermore, FOXP3 is transiently upregulated in Teff cells upon TCR-activation without endowing them with suppressive function (3, 4), thus blurring the distinction between human Treg and activated Teff cells.

Using a single-cell cloning strategy that allows the discrimination between activated Teff contaminants and *bona fide* FOXP3-expressing Treg clones, we have recently shown that the human FOXP3^+^ Treg population is functionally heterogeneous, containing a sizeable proportion of clones with an impaired capacity to suppress the proliferation of Teff cells despite exhibiting the hallmark surface phenotype of functional Treg cells (5, 6). We have further demonstrated that this FOXP3-positive, suppression-negative (FPSN) subpopulation, resembles its FOXP3-positive, suppression-positive (FPSP) counterpart in the demethylation status of the Treg-specific demethylated region (TSDR) of the *FOXP3* locus, as well as in the global Treg gene expression signature (6). These findings indicated that these non-suppressive FOXP3^+^ cells likely originate from previously functional Treg cells. There are currently no markers to delineate these dysfunctional FOXP3^+^ cells, and their prevalence and potential role in autoimmunity remains unknown. This study aims to characterize the factors that drive loss of suppressive function in human Treg cells, and to identify surface markers of dysfunctional Treg cells.

Several inflammatory mediators have been shown to modulate the function of Treg cells, including inflammatory cytokines such IL-1β, TNF-α, and IL-6, as well as several TLR ligands and microbial metabolites [reviewed in (7)]. The effects of IL-6 on Treg function have been particularly well-studied. IL-6 plays a critical role in regulating the balance between T helper 17 (Th17) cells and Treg cells, by favoring the differentiation of Th17 cells over Treg cells in the presence of TGF-β (8, 9). IL-6 has also been shown to inhibit *in vitro* and *in vivo* Treg-mediated suppression in mice (10–12) and humans (13). Clinically, elevated circulating levels of IL-6 are detected in the sera and urine of SLE patients, and correlate with disease severity (14). IL-6 is also highly elevated in the synovia of RA patients (15), and in the intestinal mucosa of inflammatory bowel disease (IBD) patients (16). Blockade of IL-6 using tocilizumab, an approved treatment for RA and other autoimmune disorders, has been shown to correlate with increased frequency of Treg cells, although Treg function was not assessed in these settings (17–20).

IL-6 signals through a receptor complex comprised of IL-6R (CD126) and gp130 (CD130) (21). Gp130 is part of the receptor complex for several cytokines, including IL-6, IL-27, IL-11, Leukemia Inhibitory Factor (LIF), Oncostatin M (OSM), Ciliary Neurotrophic Factor (CNTF), Cardiotrophin 1 (CT-1), and Cardiotrophin-like Cytokine (CLC) (22). The gp130 receptor is ubiquitously expressed on hematopoietic and non-hematopoietic cells, and its deletion in mice is embryonically lethal due to defects in cardiac development (23). However, postnatal conditional abrogation of gp130 in hematopoietic cells results in impaired lymphocyte development (24).

IL-27 is a cytokine of the IL-12 family. It is a heterodimer composed of the IL-27p28 and the Epstein-Barr virus induced 3 (Ebi3) subunits, and is produced by activated antigen-presenting cells (APC) such as dendritic cells and macrophages (25). IL-27 signals through the IL-27 receptor complex comprised the IL-27RA (WSX-1) and gp130 (25). Both pro- and anti-inflammatory roles have been described for IL-27. As a pro-inflammatory cytokine, IL-27 has been shown to induce the production of IFN-γ and favor the differentiation of Th1 cells in a STAT1-dependent manner (26–28). Furthermore, IL-27 interferes with TGFβ-induced generation of Treg cells (29), and more recently, Zhu et al. reported that IL-27, delivered using an adeno-associated virus (AAV)-based system results in a rapid depletion of Treg cells and enhances anti-tumor responses in a mouse model of melanoma (30). On the other hand, IL-27 has been shown to increase the production of IL-10 by effector CD4^+^ and Tr1 cells (31–33), and to attenuate Th17-mediated inflammation in the EAE model (33–35). Furthermore, some groups have reported a paradoxical role for IL-27 in potentiating the suppressive function of Treg cells (36).

In this study, we investigated factors that drive loss of suppressive function in human FOXP3^+^ Treg cells. We found that expression of gp130 identifies Treg cells with reduced suppressive function directly *ex vivo*. Furthermore, we show that IL-6- and IL-27-mediated signaling through gp130 impairs the suppressive capacity of Treg cells. These results highlight the important role of gp130-signaling in modulating the suppressive function of human Treg cells and present a novel target for the therapeutic modulation of Treg function.

## Materials and Methods

### Donors and Cell Isolation

Peripheral blood mononuclear cells (PBMC) were purified from buffy coats of healthy donors (Sanguine Biosciences) using Ficoll-Paque PLUS density gradient (GE Healthcare), and were cryopreserved.

### Reagents

Cryopreserved PBMCs were thawed and stained with viability dye (eFluor 780; eBioscience). Antibodies against CD4 (FITC or V500), CD25 (APC), CD45RA (Alexa Fluor 700)(BD Biosciences), CD127 (PE-eFluor 610), FOXP3 (PE), TIGIT (PerCP-eFluor710) (eBioscience), Helios (Pacific Blue; Biolegend). Purified anti-FCRL3 antibody was provided by Nagata (37), and was detected with F(ab′)2 anti-mouse IgG (PE-Cy7; eBioscience). Flow cytometry analysis was performed on an LSR Fortessa analyzer (BD Biosciences), and sorting throughout this study was performed on a FACS Fusion cell sorter (BD Biosciences). Recombinant human IL-6, IL-27, CLC, IL-11 (R&D systems), and LIF (Peprotech) were added to suppression assays where indicated at the time of activation.

### Generation of Primary CD4^+^ T Clones From Human PBMC

Primary CD4^+^ clones were generated from healthy donors by single-cell sorting of CD25^High^ and CD25^Neg^ cells as described previously (5, 6). The clones were stimulated with soluble anti-CD3 (30 ng/mL; eBioscience), recombinant human IL-2 (200 U/mL) and irradiated human PBMCs as feeders, and propagated in Xvivo-15 medium (Lonza) supplemented with 5% FBS (Sigma-Aldrich). Fresh medium and IL-2 was added on day 5 and every 2 days thereafter, and clones were passaged as required. Clones were re-stimulated on day 11–12 and further expanded until harvest on day 22–24. Phenotypic analysis and functional assessment of suppressive capacity were performed in parallel.

### *In vitro* Suppression Assays

Suppression assays were performed as previously described (5). Briefly, responding allogeneic CD4^+^CD25^−^ cells (Teff) were FACS-sorted, stained with the CFSE proliferation dye (5 μM; Sigma-Aldrich) and plated at 8,000 cells/well in U-bottom 96-well plates (Sarstedt) with irradiated PBMCs as feeders (APCs) (30,000 cells/well). Treg cells were added to the culture at a ratio of 1:1 and the assays were stimulated with soluble anti-CD3 (30 ng/mL; eBioscience) for 4 days. In bead-stimulated suppression assays, cells were stimulated with anti-CD3/anti-CD28-coated beads at a 1:2 beads/cell ratio. APC-derived supernatants were generated from total healthy PBMCs stimulated separately with anti-CD3/anti-CD28-coated beads at a 1:2 beads/cell ratio. APC-derived supernatants were collected every 24 h for 3 days, and added immediately to the Treg-Teff co-culture.

Suppression values in all suppression assays were calculated based on the division index of Teff cells cultured in the absence of Treg cells using the following formula:

$$\begin{array}{l} \left(1-\left(\frac{Division\;index\;of\;Teff\;cultured\;in\;the\;presnece\;of\;Treg\;cells}{Division\;index\;of\;Teff\;cultured\;in\;the\;absence\;of\;Treg\;cells}\right)\right)*100 \end{array}$$

Where indicated, recombinant human IL-6, IL-11, IL-27, CLC (all at 1-100 ng/mL), or LIF (100–1,000 ng/mL) were added to the suppression assay at the time of activation. When cytokines were added to a suppression assay, suppression at a specific cytokine dose was calculated based on the Teff cells culture in the absence of Treg cells in the presence of the corresponding cytokine at the corresponding dose.

### Statistical Analysis

Statistical analysis was performed using the GraphPad Prism 6.0 software. One-way ANOVA, followed by multiple-comparison testing was used to compare multiple (>2) groups, and the student *t-*test were used where indicated for two-group comparisons. A *p*-value of < 0.05 was considered significant.

## Results

### APC-Derived Factors Drive the Loss of Suppressive Function in Primary Human Treg Clones

**Figure 1:**
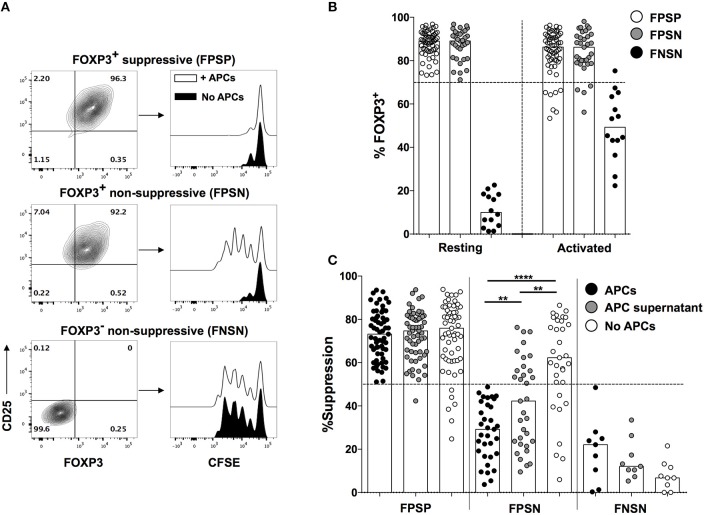
Loss of suppressive function in FOXP3^+^ Treg cells is driven by APC-derived factors. Primary FOXP3^+^ clones were generated from healthy donors as described above, and were analyzed for their ability to suppress allogeneic CD4^+^CD25^−^ Teff cells in a 1:1 Treg:Teff suppression assay in the presence or absence of irradiated PBMCs (APCs). **(A)** Representative FACS plots showing CD25 and FOXP3 expression in representative FPSP, FPSN, and FNSN clones and their suppressive function in the presence or absence of APCs. **(B)** FOXP3 expression in FPSP, FPSN, and FNSN clones before, or 48 h after re-stimulation with anti-CD3 and irradiated APCs. **(C)** Suppressive potency of FOXP3^+^ and FOXP3^−^ clones in the presence or absence of APCs, or APC-derived supernatants. Shown are the suppression values for 61 FPSP, 31 FPSN, and 9 FNSN clones from one representative experiment of three different experiments where clones were generated from three different healthy individuals. Statistical analysis was done with the one-way ANOVA followed by the Tukey post-test. **p < 0.01, ****p < 0.0001.

Although stable FOXP3 expression is considered a specific feature of Treg cells, it is now established that human CD4^+^ Teff cells can express FOXP3 upon activation, making them indistinguishable from Treg cells in activation or inflammatory contexts (3, 4). This activation-induced FOXP3 expression is transient and subsides within a few days of activation. To circumvent this issue, we have developed and exploited a single cell cloning strategy to pinpoint the phenotypic and functional status of individual cells in the heterogeneous FOXP3 expressing population. In this approach, we expand clones generated from single CD4^+^CD25^High^ and CD4^+^CD25^Neg^ primary T cells from human PBMC, and analyze their phenotypic and functional profiles at the end of a short-term activation cycle, thus allowing activation-induced FOXP3 expression to subside resulting in a state of immune quiescence. At the time of harvest, only Treg cell-derived clones maintain high FOXP3 levels, thus reliably eliminating contaminating Teff cells. Using this approach, we have previously shown that Treg clones harbor a population that is functionally impaired despite exhibiting the canonical phenotype of Treg cells including high and stable expression of FOXP3 (Figures 1A,B) (5, 6).

All assessments of suppressive capacity of FOXP3^+^ clones were thus far performed in the presence of irradiated PBMCs as APC to provide co-stimulation in the suppression assay (5, 6). APCs are a major source of several inflammatory cytokines, some of which have been shown to alter the function of mouse and human Treg cells (7). Therefore, we asked whether the loss of suppressive function observed in FPSN clones is driven by inflammatory factors provided by the APCs. To assess this hypothesis, we examined the suppressive capacity of FOXP3^+^ Treg clones in the presence or absence of irradiated APCs. Interestingly, the lack of APCs in the co-culture almost completely rescued the suppressive function of FPSN clones (Figure 1C). The absence of APCs had no significant effect on the suppressive potency of the already suppressive FPSP clones, nor did it affect the lack of suppression in control FNSN clones (Figure 1C). To determine the nature of the APC-derived factors driving the observed Treg dysfunction, we examined the suppressive capacity of Treg clones in the presence of purified supernatant of activated APCs. APC-derived supernatant was sufficient to cause a significant reduction in the suppressive potency of FOXP3^+^ clones (Figure 1C). These data demonstrate that soluble APC-derived factors contribute to the lack of suppressive function associated with FPSN clones.

### Gp130, a Component of the IL-6R Complex, Is Preferentially Expressed in Non-suppressive FOXP3^+^ Treg Cells

**Figure 2:**
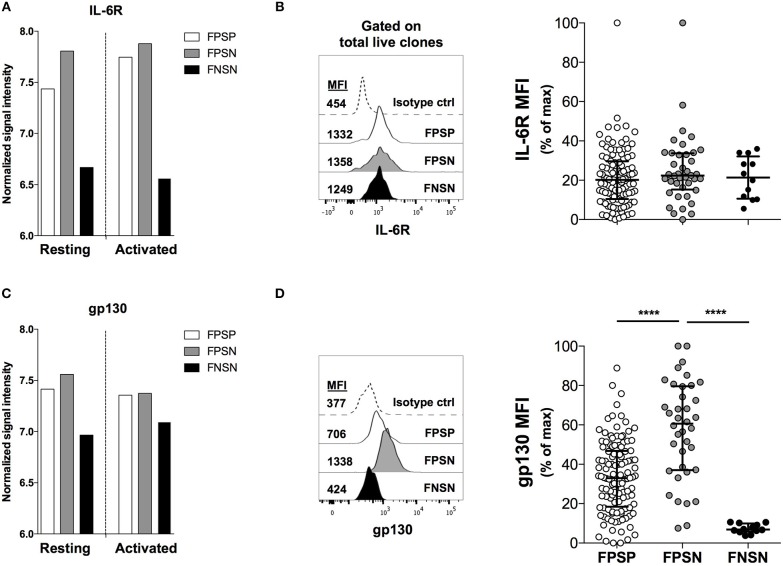
Gp130, a component of the IL-6R complex, is preferentially expressed in non-suppressive FOXP3^+^ clones. Primary FOXP3^+^ clones were generated from healthy donors as described above. FPSP, FPSN, and FNSN clones were identified based on their ability to suppress allogeneic CD4^+^CD25^−^ Teff cells in a 1:1 Treg:Teff suppression assay in the presence of irradiated APCs. Shown are the relative mRNA expression levels of the IL-6 receptor chains IL-6R **(A)** and gp130 (IL-6ST) **(C)** in FPSP, FPSN, and FNSN clones as measured at the resting state at harvest or 24 h after activation (6). **(B,D)** show the protein expression levels of IL-6R **(B)** and gp130 **(D)** on FPSP, FPSN, and FNSN clones at the time of harvest. Data were normalized using the internal normalization feature in GraphPad Prism where the largest value in the data was set to 100 and the lowest value was set to 0. Shown are the results from one representative experiment of six different experiments where clones were generated from six different healthy individuals. Statistical analysis was done with the one-way ANOVA followed by the Tukey post-test. ****p < 0.0001.

We have previously performed a whole-genome expression analysis on FPSP, FPSN, and FNSN clones in order to identify gene products that distinguish human Treg cells from activated Teff cells and further identify the different functional subpopulations of Treg cells (6). Examining the expression levels of inflammatory cytokine receptors on the three populations, we observed an increased transcription level of the IL-6 receptor subunit (IL-6R) mRNA on both FOXP3^+^ Treg subpopulations relative to FOXP3^−^ controls in resting and activated conditions (Figure 2A). Although we did not observe a significant difference in IL-6R mRNA expression between FPSP and FPSN clones, we were prompted to further investigate the IL-6 pathway in the Treg clones due to the well-established role of IL-6 as a major antagonist of Treg function (10–13, 38). To that end, we generated FOXP3^+^ and FOXP3^−^ clones from CD4^+^CD25^High^ and CD4^+^CD25^−^ cells, respectively, and identified FPSP, FPSN, and FNSN clones by examining their suppressive capacity in the presence of irradiated APCs. In parallel, we assessed the expression of IL-6R on the three subsets by flow cytometry prior to activation. Most examined clones expressed IL-6R, although no significant differences in IL-6R protein expression were observed among the three subsets (Figure 2B). Given the possibility of IL-6 trans-signaling in cells that do not express IL-6R (39, 40), we examined the expression levels of gp130 whose surface expression is required for IL-6 signaling. Interestingly, despite its mRNA being equally expressed by the three populations (Figure 2C), gp130 protein was significantly elevated on the surface of FPSN clones compared to FPSP and FNSN clones (Figure 2D), suggesting a potential role for this cytokine receptor in the functional impairment of the FPSN subset.

### Gp130-Expressing Treg Cells Are Enriched Amongst the Naïve T Cell Compartment, and Display Features of Functional Instability

**Figure 3:**
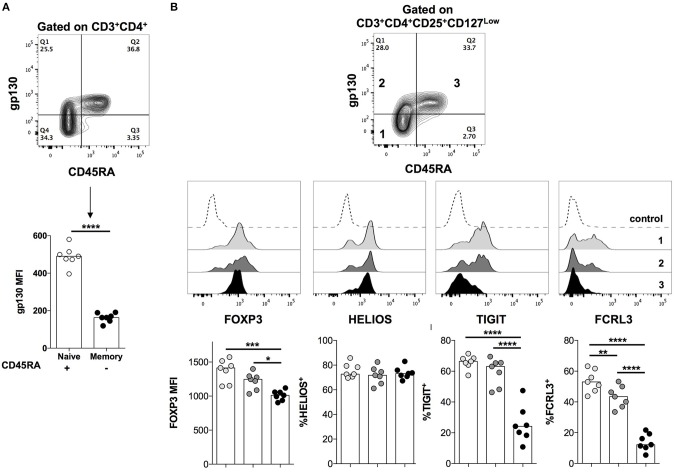
Gp130 is preferentially expressed on naïve CD4^+^ T cells. PBMCs were isolated from healthy individuals and analyzed *ex vivo* by flow cytometry for the expression of the indicated markers. **(A)** The expression of gp130 on naïve vs. memory CD4^+^ T cells. Statistical analysis was performed using the student *t-*test. **(B)** The expression of gp130 on naïve vs. memory Treg cells and its correlation with the expression of FOXP3, Helios, TIGIT, and FCRL3. Shown are the combined results from seven different healthy individuals. Statistical analysis was done with the one-way ANOVA followed by the Tukey post-test. *p < 0.05, **p < 0.01, ***p < 0.001, ****p < 0.0001.

We next assessed the *ex vivo* expression of gp130 on total CD4^+^ T cells and in Treg cells in healthy individuals. We found that gp130 is preferentially expressed on predominantly naïve (CD45RA^+^) CD4^+^ T cells, while the memory (CD45RA^−^) compartment contains both gp130^High^ and gp130^Low^ cells (Figure 3A). A similar pattern of gp130 expression was observed in CD45RA^+^ vs. CD45RA^−^ Treg (CD4^+^CD25^+^CD127^Low^) cells (Figure 3B). We have previously reported that Helios expression is associated with enhanced suppressive capacity and maximal repression of inflammatory cytokine production by human Treg cells (6). Moreover, we recently identified two surface proteins, TIGIT and FCRL3, as a reliable marker combination that distinguishes Helios^+^ from Helios^−^ Treg cells in human peripheral blood (6). We then investigated the expression of these novel surface markers in relation to gp130. Since both TIGIT and FCRL3 are preferentially expressed on memory Treg cells (6), while gp130 is highly expressed on CD45RA^+^ Treg cells, we analyzed the expression of these markers on naïve and memory Treg subpopulations, identified based on CD45RA expression, and further distinguished based on gp130 expression (Figure 3B). As previously reported, FOXP3 expression is lower in naïve Treg cells relative to memory Treg cells (41). At the steady state, we did not observe a significant difference in FOXP3, Helios or TIGIT protein expression levels between gp130^High^ and gp130^Low^ within the memory Treg subset (Figure 3B). However, the frequency of FCRL3^+^ cells is significantly reduced within the memory gp130^High^ Treg cells (Figure 3B). Thus, given the correlation between FCRL3 expression and stable Treg cell function (6), this reduced expression of FCRL3 could be indicative of reduced stability of gp130^High^ Treg cells.

### *Ex vivo* gp130 Expression Identifies Treg Cells With Reduced Suppressive Capacity

**Figure 4:**
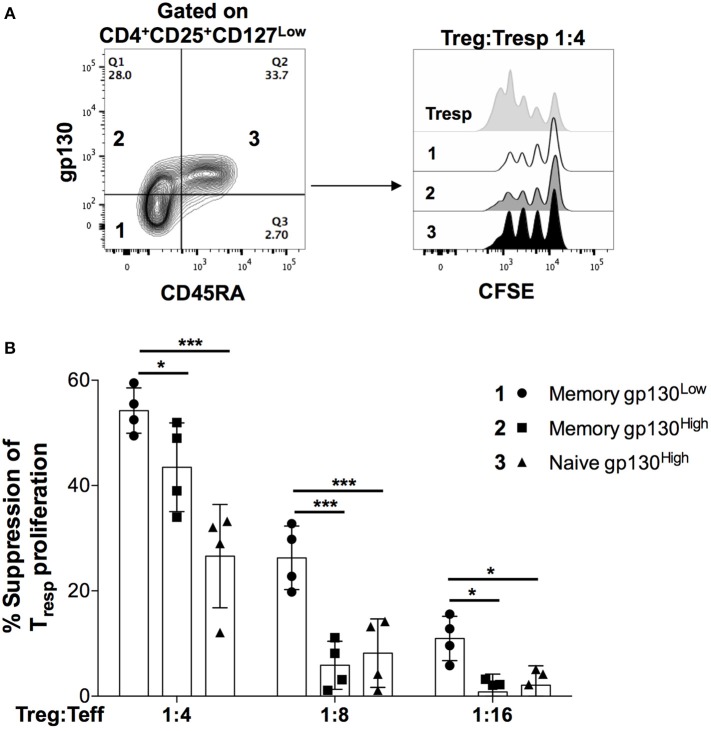
High gp130 expression identifies Treg cells with reduced suppressive capacity *ex vivo*. Naïve and memory Treg cells (CD4^+^CD25^High^CD127^Low^) cells were FACS-sorted according to their gp130 expression levels and co-cultured with CFSE-labeled, FACS-sorted CD4^+^CD25^−^ Teff cells in the presence of anti-CD3 and irradiated PBMCs for 96 h. Shown are representative FACS plots **(A)** and the percentage of suppression **(B)** of Teff cells at multiple Treg:Teff ratios in two different experiments on two different healthy donors. Statistical analysis was done with the one-way ANOVA followed by the Dunnett post-test. *p < 0.05, ***p < 0.001.

We next sought to examine the correlation between gp130 expression and suppressive function of Treg cells directly *ex vivo*. To that end, we FACS-sorted the following 3 subpopulations of CD4^+^CD25^High^CD127^Low^ Treg cells: (1) CD45RA^−^gp130^Low^, (2) CD45RA^−^gp130^High^, and (3) CD45RA^+^gp130^High^. Given the high expression of gp130 on all naïve CD4^+^ T cells, the fourth subpopulation CD45RA^+^gp130^Low^ is almost non-existent and, therefore, we did not include it in the analysis (Figure 4A). Within the memory Treg population, we found that the suppressive potency of memory gp130^High^ cells was significantly lower than that of gp130^Low^ cells, and was comparable to that of naïve Treg cells, which have previously been reported to have a greatly reduced suppressive capacity in comparison with memory Treg cells (Figure 4B) (41). These results confirm our findings in Treg clones that gp130 identifies Treg cells with reduced suppressive capacity, and suggest that gp130 likely transmits inflammatory signals that dampen the suppressive capability of Treg cells.

### IL-6 and IL-27 Inhibit the Suppressive Function of FOXP3^+^ Treg Cells *ex vivo*

**Figure 5:**
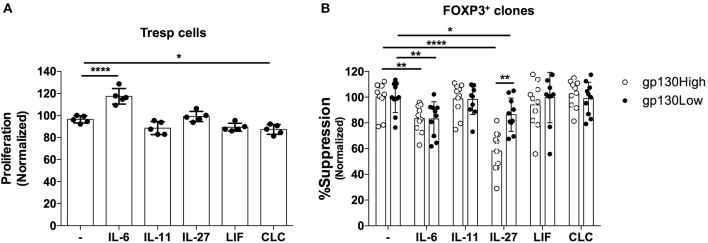
Effect of gp130-binding cytokines on the suppressive activity of primary FOXP3^+^ T cell clones. FOXP3^+^ clones were generated from healthy donors and assessed for their ability to suppress allogeneic CD4^+^CD25- Teff cells in a 1:1 Treg:Teff suppression assay in the absence of irradiated APCs. **(A)** The effects IL-6 (50 ng/mL), IL-11 (100 ng/mL), IL-27 (20 ng/mL), LIF (100 ng/mL), and CLC (1 ug/mL) on the proliferative response of Teff cells activated in the absence of clones. **(B)** The effects of the indicated cytokines on the suppressive potency of gp130^High^ (gp130 MFI > 600) vs. gp130^Low^ (gp130 MFI <500) FOXP3^+^ clones identified based on their gp130 expression levels before activation. Shown are the results from one representative experiment of three different experiments where clones were generated from three different healthy individuals. Statistical analysis was done with the one-way ANOVA followed by the Dunnett post-test. *p < 0.05, **p < 0.01, ****p < 0.0001.

Several cytokines utilize gp130 as part of their receptor complexes, activating various downstream signaling pathways and driving different biological processes (42). Here we sought to identify gp130-signaling cytokines that alter the suppressive function of Treg cells. We generated primary Treg clones and assessed their capacity to suppress the proliferation of Teff cells in the presence of the gp130-signaling cytokines IL-11, LIF or CLC, IL-6, and IL-27. We first analyzed the effects of these cytokines on the proliferative capacity of responder Teff cells TCR-activated in the absence of Treg cells. We observed that only IL-6, but not the other tested cytokines, significantly increased the proliferation of Teff cells (Figure 5A). We next examined the suppressive potency of Treg clones in the absence or presence of exogenous cytokines. While the suppressive potency of Treg clones was not altered upon the addition of exogenous IL-11, LIF or CLC, both IL-6, and IL-27 markedly decreased Treg-mediated suppression of Teff cell proliferation (Figure 5B). However, while IL-27 exhibits a significantly higher modulatory effect on gp130^High^ relative to gp130^Low^ clones, IL-6 alters the suppressive function of gp130^High^ and gp130^Low^ clones to a similar extent, suggesting that IL-6 may additionally alter Treg-mediated suppression through the enhancement of Teff proliferation (Figure 5A).

**Figure 6:**
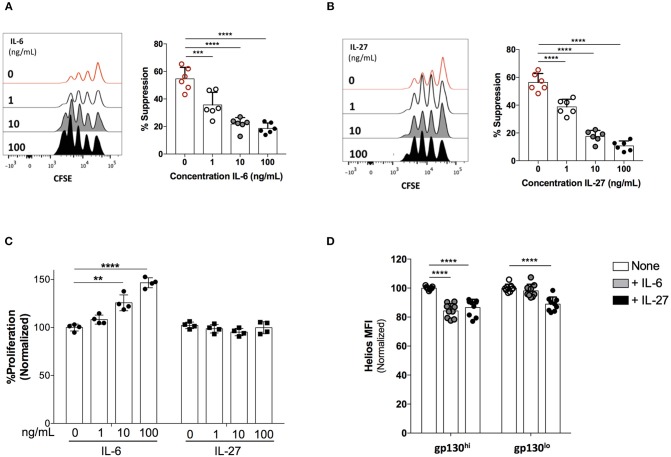
IL-6 and IL-27 inhibit Treg cell suppressive function *ex vivo*. Treg cells (CD4^+^CD25^High^CD127^Low^) cells were FACS-sorted and co-cultured with CFSE-labeled, FACS-sorted CD4^+^CD25^−^ Teff cells in the presence exogenous IL-6 or IL-27. Cells were stimulated with human αCD3/αCD28 coated beads (Treg Suppression Inspector; MACS Miltenyl Biotec) at a 1:2 bead: cell ratio for 96 h. Treg and Teff were plated at a ratio of 0:1 or 1:8, **(A)** The effects of rhIL-6 on the suppressive function of Treg cells. **(B)** The effects of rhIL-27 on the suppressive function of Treg cells. **(C)** The impact of IL-6 and IL-27 on the proliferative response of Teff cells activated in the absence of Treg cells. **(D)** Gp130^High^ and gp130^Low^ Treg cells (CD4^+^CD25^+^CD127^Low^) were FACS-sorted and stimulated with human αCD3/αCD28 coated beads for 48 h in the presence of CD4^+^CD25^−^ Teff cells at a 1:4 Treg:Teff ratio. Where indicated, rIL-6 (10 ng/mL) or rIL-27 (10 ng/mL) was added. Helios expression was assessed by flow cytometry. Data are representative of at least three independent experiments performed on cells isolated from different healthy individuals. Statistical analysis was done with the one-way ANOVA followed by the Dunnett post-test. **p < 0.01, ***p < 0.001, ****p < 0.0001.

We next assessed the impact of exogenous IL-6 and IL-27 on the suppressive function of Treg cells *ex vivo*. To that end, we sorted CD4^+^CD25^+^CD127^low^ Treg cells and measured their capacity to suppress the proliferation of TCR-activated CD4^+^CD25^−^ Teff cells in the presence of titrated amounts of IL-6 or IL-27. Following a 96-h incubation period, Teff cell proliferation was assessed as a measure of Treg cell suppressive capacity. Both IL-6 (Figure 6A) and IL-27 (Figure 6B) significantly reduced the suppressive function of Treg cells in a dose-dependent manner (Figure 6A). While IL-6 treatment also caused a significant increase in the proliferation of Teff cells in the absence of Treg cells, IL-27 did not directly alter Teff proliferation (Figure 6C). These data suggest that while both IL-6 and IL-27 inhibit the suppressive function of human Treg cells, IL-27 likely acts directly on Treg cells while IL-6 also indirectly impairs Treg function through enhancement of Teff cell proliferation.

Finally, we and others have previously described a key role for the Helios transcription in the functional stability of Treg cells (6, 43–45). We questioned whether IL-6 and IL-27-mediated loss of Treg suppressive function is associated with alterations in Helios expression. Indeed, we observed that both IL-6 and IL-27 cause a significant reduction in the levels of Helios expression in human Treg cells co-cultured with Teff cells (Figure 6D), suggesting that gp130 signaling, either directly or indirectly, could impact the regulatory network of Helios leading to impaired suppression by Treg cells. Further work is needed to elucidate the exact molecular mechanisms through which these gp130-signaling cytokines affect Helios expression and Treg function.

### Blockade of the gp130 Receptor Augments Treg Cell Suppressive Activity

As both IL-6 and IL-27 signal through gp130 and abrogate Treg cell suppression, we next determined whether functional blockade of gp130 could rescue Treg cell suppressive activity. To achieve this, we made use of a recently described small molecule inhibitor of gp130, LMT-28, shown to potently inhibit gp130-mediated signaling (46). We first assessed the potency of the gp130 inhibitor in preventing gp130-mediated cytokine signals in T cells by evaluating STAT3 phosphorylation.

**Figure 7:**
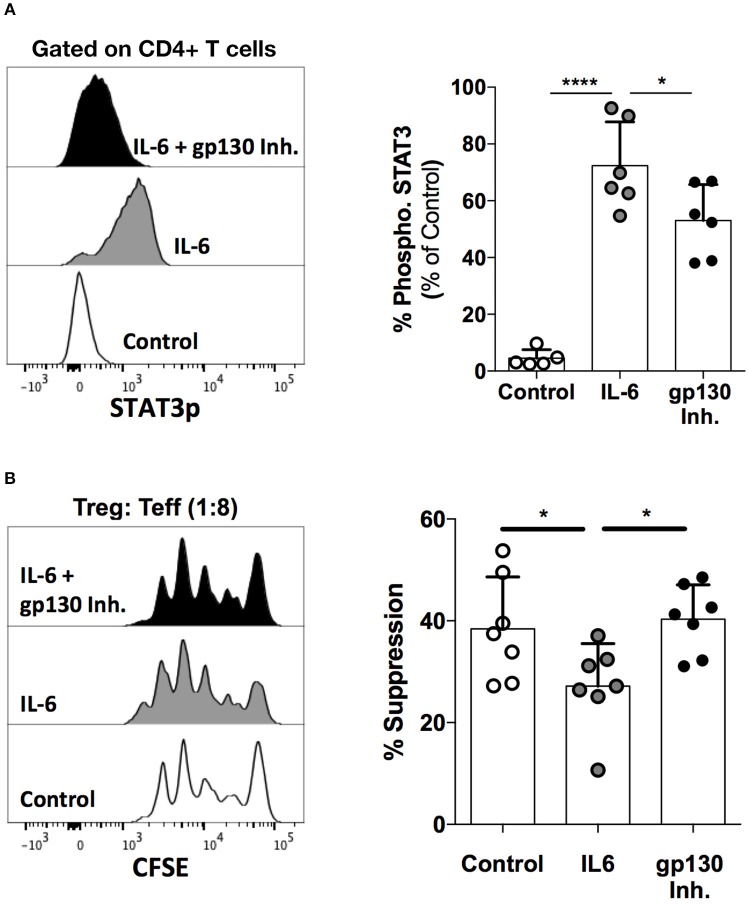
Functional blockade of gp130 augments Treg cell function. **(A)** Healthy human PBMCs were incubated with the gp130 inhibitor LMT-28 (10 μM) for 1 h, followed by stimulation with rhIL-6 (10 ng/mL) for 10 min. STAT3p expression was evaluated using multi-parametric flow cytometry. Shown is STAT3 expression in gated CD4^+^ T cells. **(B)** Naive Treg (CD4^+^CD45RA^+^CD25^+^CD127^Low^) cells were sorted from healthy human PBMCs and co-cultured with CD25^−^ Teff cells in the presence of irradiated PBMCs and soluble anti-CD3 (30 ng/mL) for 96 h. Cells were plated at a 0:1 or 1:8 Treg: Teff cell ratio along with rhIL-6 (10 ng/mL) with or without LMT-28 (10 μM). Shown data is representative of 3 independent experiments. Statistical analysis was done with the one-way ANOVA followed by the Dunnett post-test. *p < 0.05, ****p < 0.0001.

IL-6-mediated signaling through gp130 activates STAT3, which is subsequently phosphorylated. In order to test the potency of the gp130 inhibitor, we evaluated STAT3 phosphorylation in the presence and absence of the drug in healthy human PBMCs stimulated with IL-6. Total healthy PBMCs were plated with the inhibitor for 1 h followed by stimulation with IL-6 for 10 min, after which STAT3 phosphorylation was evaluated using multi-parametric flow cytometry. We show that the gp130 inhibitor significantly abrogates IL-6-mediated STAT3 phosphorylation in CD4^+^ T cells (Figure 7A).

Finally, we sought to determine whether gp130 inhibition could antagonize the effect of IL-6 signaling on the suppressive function of Treg cells. Indeed, while the suppressive function of naïve (CD45RA^+^) Treg cells is significantly reduced by IL-6, treatment with the gp130 inhibitor is able to restore the suppressive capacity of Treg cells to normal levels (Figure 7B). These data further highlight the role of the gp130 axis in the regulation of Treg function, and demonstrate that gp130 may present a viable therapeutic target for the modulation of Treg activity.

## Discussion

We have previously reported on the remarkable degree of functional heterogeneity within human FOXP3^+^ Treg cells. In particular, we showed that CD4^+^CD25^High/Bright^ Treg cells, albeit highly enriched in suppressive FOXP3^+^ T cells, harbor a pool of *bona fide* FOXP3^+^ Treg cells with compromised suppressive function, despite the maintenance of hallmark phenotypic and functional T_reg_ features. Notably, these non-suppressive Treg cells are indistinguishable from functionally suppressive Treg cells using the conventional markers of human Treg cells. In this study, we sought to identify potential factors that underlie such loss of function in FOXP3^+^ cells. We demonstrated that APC-derived factors are responsible for the observed functional impairment, and identified gp130 as a surface receptor that is highly expressed in non-suppressive FOXP3^+^ clones. We further showed that IL-6 and IL-27 negatively modulate Treg function. These results highlight the role of gp130 in regulating the function of human Treg cells and propose modulation of gp130 function as a potential therapeutic avenue for regulation of human Treg function in various disease settings.

Previous studies have associated IL-27 with both pro-inflammatory and anti-inflammatory roles in several animal models. As a pro-inflammatory cytokine, IL-27 signaling is a potent inducer of T-bet and IFN-γ (26–28), and plays a critical role in mediating CD4^+^ T cell responses against chronic lymphocytic choriomeningitis virus (LCMV) infection (47). Moreover, in a model of helminth-induced inflammatory bowel disease (IBD), *Il27ra* deficiency impaired Th1 responses in the intestine resulting in inefficient worm expulsion and delayed onset of colitis (48). IL-27 has also been shown to inhibit the TGFβ-mediated induction of Treg cells (29), and adoptive transfer of *Il27ra*^−/−^ Treg cells into lymphopenic mice resulted in attenuated colitis, which was attributed to increased induction of peripheral Treg cells (49). Furthermore, transgenic mice overexpressing IL-27 succumbed to spontaneous inflammation associated with a severe diminishment of their Treg pool (50). More recently, Zhu et al. reported that systemic delivery of IL-27 resulted in a rapid depletion of Treg cells and enhanced T cell-mediated inhibition of tumor growth in a melanoma mouse model (30). In contrast, a study by Do et al. reported that *Il27ra*^−/−^ Treg cells are defective in their suppressive capacity and are unable to suppress inflammation in a colitis model (36). The authors further reported that stimulation of Treg cells in the presence of IL-27 substantially improved the suppressive function of Treg cells *in vitro* and *in vivo* (36). These seemingly contrasting results could be a result of the different mouse genetic backgrounds used in the aforementioned studies, C57BL/6 and BALB/c (30, 36, 49). Furthermore, it is becoming increasingly appreciated that differences in the composition of commensal microbiota have a significant influence on immune responses and disease outcome even within animals of the same strains (51), and therefore the influence of microbial composition in animal models on the these seemingly contrasting findings cannot be ruled out.

It should be noted that the majority of the studies that examined the *in vivo* effects of IL-27 have used *Il27ra*^−/−^ mice. However, IL-27 may not be the only ligand for IL-27RA. Indeed, a recent study by Wang et al. has demonstrated that IL-35 also signals through a receptor complex involving the IL-27RA (52). IL-35 is an anti-inflammatory cytokine expressed by a suppressive subset of mouse T cells termed iTr35 (53, 54). IL-35 also shares the Ebi3 with IL-27 (53, 54). Thus, the shared nature of the IL-27RA complicates the interpretation of studies that relied on *Il27ra*^−/−^ mice to examine the role of IL-27 *in vivo*.

Interestingly, Do et al. have also examined the effects of IL-27 on the suppressive function of human Treg cells and reported that IL-27 significantly improves their suppressive function (36). While this is in contrast to our findings showing a negative impact of IL-27 on human Treg function, there are important differences in the assessment of Treg function between the two studies. While we added IL-27 at the time of activation of the suppression co-culture, Do et al. activated Treg cells separately in the presence or absence of IL-27 for 3 days prior to co-culturing with Teff cells. Furthermore, Do et al. did not report details on the levels of activation achieved in their suppression assays to allow a clear estimation of the quality and magnitude of the modulation of Treg activity achieved by IL-27 (36). Importantly, we observed no effect of IL-27 on Teff cells cultured alone as a control, indicating that our observations of reduced Treg function in the presence of IL-27 are due to its direct action on Treg cells.

The mechanism through which IL-27 acts on Treg cells is unclear. However, previous studies have shown that IL-6 inhibits Treg function through the activation of the STAT3 pathway and overcoming the FOXP3-mediated inhibition of RORγt (55). IL-27 signaling through gp130 has also been shown to activate STAT3 (56, 57) and, therefore, it is possible that the mechanism of inhibition of Treg function is shared between IL-6 and IL-27. Additionally, IL-27 activates STAT1 leading to the inhibition of IL-2 production by T cells through the activation of the suppressor of cytokine signaling 3 (SOCS3) (50, 58, 59). Furthermore, IL-27 interferes with T cell responsiveness to IL-2 (30, 58). Given the vital role played by IL-2 in the survival and function of Treg cells, this latter effect of IL-27 likely plays a significant role in modulating Treg function.

An interesting observation in our study is the restoration of suppressive capacity in non-suppressive FOXP3^+^ clones in the absence of APCs. This indicates that modulation of Treg function is largely mediated by local inflammatory factors, and is reversible, thus highlighting the resilient yet adaptable nature of human Treg cells in response to cues in the microenvironment. The significance of this functional heterogeneity is not clearly understood. One potential advantage of having subpopulations of Treg cells with differential responsiveness to inflammatory mediators would be the facilitation of a rapid down-modulation of Treg suppressive function in response to an infection. This would allow a partial relief of Treg activity and an enhanced initiation of an effective protective response. It would, therefore, be critical that down-modulated Treg cells can regain their suppressive capacity upon pathogen clearance. Whether deregulated and chronic production of inflammatory mediators can cause permanent loss of Treg function and contribute to autoimmunity remains to be investigated. There is, however, mounting evidence in mouse models indicating that Treg cells can, under inflammatory conditions such as lymphopenia or chronic infections, lose their Foxp3 expression and suppressive function and differentiate into Teff-like cells with inflammatory potential (60–62). It is not clear if these former Foxp3^+^ cells can regain Foxp3 expression and suppressive capacity in homeostatic conditions *in vivo*. Persistent FOXP3 loss in a highly inflammatory environment may cause long-lasting quantitative and qualitative defects in the Treg population.

Our study also highlights the importance of considering the influence of the inflammatory milieu on Treg cells when assessing Treg function in human autoimmune disease. Although intrinsic defects in Treg function may be a possible underlying cause of organ-specific autoimmunity, it is highly likely that Treg dysfunction could be a consequence of functional modulation by extrinsic factors that are abundant *in situ* in inflammatory conditions. Monitoring Treg cells with an increased susceptibility to functional modulation, gp130^high^ cells for instance, in autoimmune patients could provide valuable insight into the functional status of the Treg population in these patients. Regardless of whether Treg dysfunction is causative or secondary in organ-specific autoimmunity, identification of pathways that interfere with Treg function is highly needed in order to design strategies through which Treg function can either be enhanced to allow a better control of autoimmune responses, or abrogated to bolster anti-tumor responses.

## Data Availability

Publicly available datasets were analyzed in this study. This data can be found here: http://www.ncbi.nlm.nih.gov/geo/query/acc.cgi?acc=GSE65650; https://www.ncbi.nlm.nih.gov/pubmed/25762785/.

## Ethics Statement

Blood was obtained from healthy volunteers after informed consent. All samples used in this study were collected in accordance with the ethical review board of the Research Institute of the McGill University Health Center.

## Author Contributions

KB, ED'H, and CP designed the project. KB, SB, and ED'H performed the experiments and analyzed the data. KB and CP wrote the manuscript.

### Conflict of Interest Statement

The authors declare that the research was conducted in the absence of any commercial or financial relationships that could be construed as a potential conflict of interest. The reviewer MO and handling editor declared their shared affiliation at the time of review.
